# Distinct Molecular Phenotypes in Male and Female Schizophrenia Patients

**DOI:** 10.1371/journal.pone.0078729

**Published:** 2013-11-11

**Authors:** Jordan M. Ramsey, Emanuel Schwarz, Paul C. Guest, Nico J. M. van Beveren, F. Markus Leweke, Matthias Rothermundt, Bernhard Bogerts, Johann Steiner, Sabine Bahn

**Affiliations:** 1 Department of Chemical Engineering and Biotechnology, University of Cambridge, Cambridge, United Kingdom; 2 Department of Psychiatry, Erasmus University, Medical Center, Rotterdam, The Netherlands; 3 Department of Psychiatry and Psychotherapy, University of Cologne, Cologne, Germany; 4 Department of Psychiatry and Psychotherapy, Central Institute of Mental Health, Medical Faculty Mannheim, University of Heidelberg, Mannheim, Germany; 5 Department of Psychiatry, University of Muenster, Muenster, Germany; 6 Department of Psychiatry, University of Magdeburg, Magdeburg, Germany; 7 Department of Neuroscience, Erasmus University Medical Centre, Rotterdam, The Netherlands; University of Queensland, Australia

## Abstract

**Background:**

In schizophrenia, sex specific dimorphisms related to age of onset, course of illness and response to antipsychotic treatment may be mirrored by sex-related differences in the underlying molecular pathways.

**Methodology/Principal Findings:**

Here, we have carried out multiplex immunoassay profiling of sera from 4 independent cohorts of first episode antipsychotic naive schizophrenia patients (n = 133) and controls (n = 133) to identify such sex-specific illness processes in the periphery. The concentrations of 16 molecules associated with hormonal, inflammation and growth factor pathways showed significant sex differences in schizophrenia patients compared with controls. In female patients, the inflammation-related analytes alpha-1-antitrypsin, B lymphocyte chemoattractant BLC and interleukin-15 showed negative associations with positive and negative syndrome scale (PANSS) scores. In male patients, the hormones prolactin and testosterone were negatively associated with PANSS ratings. In addition, we investigated molecular changes in a subset of 33 patients before and after 6 weeks of treatment with antipsychotics and found that treatment induced sex-specific changes in the levels of testosterone, serum glutamic oxaloacetic transaminase, follicle stimulating hormone, interleukin-13 and macrophage-derived chemokine. Finally, we evaluated overlapping and distinct biomarkers in the sex-specific molecular signatures in schizophrenia, major depressive disorder and bipolar disorder.

**Conclusions/Significance:**

We propose that future studies should investigate the common and sex-specific aetiologies of schizophrenia, as the current findings suggest that different therapeutic strategies may be required for male and female patients.

## Introduction

Schizophrenia is a severe psychiatric illness with a lifetime risk between 0.5 and 1% [1]. Despite the similar prevalence in males and females, sex differences have been established for many characteristics of the disorder [2]. These include age of onset (typically younger in males), and differential symptoms, disease course and treatment response. Numerous studies have explored structural and neurophysiological sex dimorphisms of schizophrenia by looking at specific molecules in a targeted manner [2]. In particular, sex hormones have been hypothesised as playing a central role in the differences in schizophrenia between male and female patients. Estrogen has been shown to have a variety of structural, functional and molecular functions leading to protective effects in female schizophrenia patients. In line with this, psychosis has been associated with lower estrogen phases [2] and add-on estradiol treatment has been reported to be beneficial for symptom improvement [3]. Other studies have tested the idea that testosterone may have deleterious effects on brain function and the vulnerability threshold to schizophrenia, although the results of these investigations are inconclusive. In a study using animal models treated with dopamine agonists and antagonists, researchers demonstrated that treatment with estradiol, but not testosterone, led to normalisation of the behavioural effects [4]. On the other hand, an inverse correlation between testosterone levels and negative symptom severity was reported for men, and adjunctive androgen treatment in male schizophrenia patients resulted in significant improvement in negative symptoms [5].

Due to these sex-related differences, the theory has emerged that males and females with schizophrenia may be affected by different subtypes of the disorder [6]–[8]. This categorization of sex-specific schizophrenia subtypes has been performed at the clinical and developmental levels but no studies to date have attempted to characterise the underlying molecular pathways. Here, we have carried out multiplexed immunoassay profiling analyses in a multi-centre study, comprising 4 independent cohorts of 133 antipsychotic naive, first episode schizophrenia patients and 133 well-matched healthy control subjects. In addition, we used 5 cohorts comprised of 196 treated major depressive disorder (MDD) patients and 7 cohorts of 92 treated bipolar disorder (BPD) patients with matched controls to assess sex-specific molecular markers of these illnesses. The immunoassay panel consisted of assays for immune factors, hormones, growth factors, transport molecules and enzymes. This platform has been used previously to explore molecular changes in cancer, autoimmune disorders and cardiovascular diseases, as well as in neurological disorders and various psychiatric diseases such as schizophrenia [9]–[13].

The main objective of the present study was to determine whether sex-specific molecular patterns can be identified in serum of schizophrenia patients and if any of these could be related to measures of symptom severity on the Positive and Negative Syndrome Scale (PANSS). It was also of interest to determine if similar differences occurred in 33 patients who received 6 weeks of antipsychotic treatment. Finally, we examined shared and distinct sex-specific biomarkers for schizophrenia, MDD, and BPD for the same set of analytes.

## Methods

### Clinical Samples

All patients were suffering from the first illness episode and were antipsychotic naive at the time of sample collection. Samples were collected following strict standard operating procedures over a period of 3 years. Samples were collected between 8 AM and 10 AM, with no systematic differences between patients and controls. Ethics statement: the ethical committees of the participating hospitals at the Universities of Cologne, Muenster and Magdeburg each approved the protocols of the study, informed written consent was given by all participants and studies were conducted according to the Declaration of Helsinki. In cases where the participants may have had a compromised capacity or ability to give consent, next of kin, carer takers or guardians consented on their behalf. All diagnoses were carried out using the Diagnostic and Statistical Manual (DSM)-IV and clinical tests performed by psychiatrists under Good Clinical Practice-compliance to minimize variability. Symptom severity was assessed using the Positive and Negative Syndrome Scale (PANSS) rating system. Clinicians had access to all detailed clinical files including medical histories by proxy and referral letters from the general practitioners. Patients and controls with severe substance abuse and other medical conditions such as type 2 diabetes mellitus, hypertension, endocrine abnormalities, cardiovascular or autoimmune diseases were not included in the study. In addition, controls with a family history of mental illness were excluded from the study. The demographic details are shown in **Table 1**. Cohort 1 was from University of Cologne Department of Psychiatry, Germany; Cohort 2 was from University of Muenster, Germany; and Cohorts 3 and 4 were from University of Magdeburg, Germany. Males and females were matched separately between patients and controls in each cohort according to age, body mass index (BMI), waist circumference, and tobacco and cannabis consumption where this information available. There were no significant differences in these variables between patients and controls and all participants were white European. The same procedures were followed for sample collection from treated major depressive disorder (MDD) and bipolar disorder (BPD) patients and matching controls. The majority of MDD and BPD patients were not in their first episode of illness, and BPD patient type and mood state were heterogeneous. Demographic details can be found in **Tables S1 and S2 in the File S1** for MDD and BPD patients, respectively.

**Table 1:** Schizophrenia patient demographics.

	Cohort 1		Cohort 2		Cohort 3		Cohort 4		P-values	
	M	F	M	F	M	F	M	F	M	F
Patients N	23	25	34	11	14	10	8	8	–	–
Controls N	23	25	34	11	14	10	8	8	–	–
Patients age	26.4±6	30.4±9	27.6±9	23.5±7	28.5±9	36.2±14	33.3±11	37.0±12	0.81	0.75
Controls age	27.1±6	30.2±7	27.6±8	23.6±7	29.6±9	35.6±14	32.0±9	34.3±10		
Patients BMI	22.8±2	23.1±5	22.7±2	20.9±2	23.7±4	25.3±5	22.2±2	21.2±3	0.55	0.67
Controls BMI	22.7±2	22.3±4			24.5±5	24.9±4	23.2±1	22.0±4		
Patients tobacco	13/7/3	8/8/9	15/15/4	1/10/0	9/5/0	7/3/0	4/4/0	2/5/1	0.13	0.17
Controls tobacco	11/12/0	11/14/0			6/8/0	2/8/0	3/5/0	2/6/0		
Patients cannabis	12/9/2	13/6/6	14/16/4	1/10/0	0/14/0	0/10/0	0/7/1	0/8/0	1.0	0.63
Controls cannabis	13/8/2	12/12/1			0/14/0	0/10/0	0/8/0	0/8/0		
PANSS P1-P7	23.4±5	23.3±6	17.2±6	18.0±10	22.4±7	18.8±6	14.4±5	23.9±9	–	–
PANSS N1-N7	24.5±9	22.7±7	17.7±7	17.4±9	20.1±10	18.3±9	14.9±2	16.4±5	–	–
PANSS G1-G16	49.0±9	50.0±10	37.6±10	36.6±13	44.3±14	40.1±9	33.4±6	40.8±15		
Total PANSS	96.9±19	96.0±19	72.5±20	72.0±30	86.9±27	77.2±15	62.6±11	81.0±25	–	–

Values are shown as mean ± sd. Tobacco and cannabis use is displayed as (yes/no/NA) and age is in years. M = male. F = female. N = number. BMI = body mass index (kg/m^2^). PANSS = Positive and Negative Syndrome Scale. The final two columns show that there are no significant differences in age, BMI, smoking, or cannabis use between schizophrenia patients and controls in the final combined cohort.

To investigate effects of antipsychotic treatment on biomarker candidates, 33 patients from cohort 3 were also assessed before and after a 6-week inpatient treatment period (**Table 2**). Male and female patients tested in this phase of the study showed no significant differences in age, BMI, smoking, cannabis use, or PANSS scores before treatment. Blood samples were collected from all subjects by venous puncture into S-Monovette 7.5 mL serum tubes (Sarstedt; Numbrecht, Germany). Serum was prepared using standard protocols by leaving samples at room temperature for 2 hours to allow blood coagulation, followed by centrifugation at 4,000×g for 5 minutes to remove particulate material. The resulting supernatants were stored at −80°C in LoBind Eppendorf tubes (Hamburg, Germany) prior to analysis.

**Table 2:** Demographics for treated schizophrenia cohort.

	Antipsychotic follow-up			
	M		F	
Patients N	22		11	
Patients age	29.2±8		34.6±14	
Patients BMI	23.5±4		25.0±5	
Patients tobacco	16/22/0		8/3/0	
Patients cannabis	8/14/0		1/10/0	
	**T0**	**T6**	**T0**	**T6**
PANSS P1–P7	22.3±7	12.0±4	19.4±6	10.9±4
PANSS N1–N7	18±9	15±7	20.2±10	12.8±6
PANSS G1–G16	42.7±12	28.5±9	42.3±11	27.4±9
Total PANSS	83.1±23	55.5±18	81.8±21	51.1±15
Antipsychotic type (olanzapine/quetiapine/risperidone/other)	0	5/3/6/8	0	3/4/3/1
Cumulative chlorpromazine units (mg)	0	17 347±14 928	0	15 254±10 657

Patients were from cohort 3 and were initially antipsychotic naïve. Values are shown as mean ± sd. Tobacco and cannabis use is displayed as (yes/no/NA) and age is in years. M = male. F = female. N = number. BMI = body mass index (kg/m^2^). PANSS = Positive and Negative Syndrome Scale. T0 = initial time point. T6 = after 6 weeks of antipsychotic treatment.

### Multiplexed Immunoassays

The protocol for the study participants, collection and storage of clinical samples, and test methods were carried out in compliance with the Standards for Reporting of Diagnostic Accuracy initiative [14]. The HumanMAP® multiplex immunoassay platform was used to screen the serum concentrations of 190 total analytes across all samples in the separate cohorts. The screening was carried out in a Clinical Laboratory Improvement Amendments-certified laboratory at Myriad-RBM (Austin, TX, USA) as described previously [13]. Samples were randomized and blinded to analysts using coded numbers until all biochemical assays were completed. Assays were calibrated using standards, raw intensity measurements converted to absolute protein concentrations by comparison to the standards, and performance was verified using quality control samples.

### Statistical Analysis

The multiplex immunoassay data were pre-processed by screening for those analytes which gave robust readings within the limits of quantitation in more than 70% of samples. This resulted in 92 proteins and 3 steroid hormones, which represented inflammation, hormonal, growth factor, cardiovascular system and metabolism-related pathways (**Table S3 in the File S1**). Shapiro-Wilk analysis showed that the majority of the values were not distributed normally. Therefore, values were log_e_-transformed to approximate normality. Outlying values for each analyte were excluded if a given concentration differed by more than 3 standard deviations from the mean after log_e_-transformation. For the different analyses described in the present study, this resulted in the removal of an average of approximately 0.7% of the values per analyte.

The data for the combined cohorts were used first to identify analytes which were present at significantly different levels in schizophrenia compared to control subjects using cohort, age, and sex as additional covariates in a linear model. Next, we calculated sex-diagnosis interactions for each analyte using the same additional covariates. In order to examine the robustness of our findings against deviations from method assumptions and effects of covariate adjustment, we also performed interaction tests using the non-parametric aligned rank transform method without using additional covariates [15], [16]. Analytes found to have significant sex-diagnosis interactions (P<0.05 and Q<0.30) were stratified by sex and re-analysed. This was repeated within each cohort to investigate the consistency of sex-specific patterns. The same procedure was used across all schizophrenia cohorts to identify potential molecular interactions between sex and positive, negative, and total PANSS scores in schizophrenia patients. Average correlation coefficients between analyte levels and scores across cohorts were found using Fisher’s z transformation [17]. The effect of treatment with antipsychotics on serum analyte levels for 33 patients was evaluated using repeated measures analysis of variance (ANOVA), with sex as a between subjects variable and adjustment for analyte values at baseline. P-values were adjusted for the false discovery rate to account for multiple comparisons and control chance findings (FDR; given as Q values) according to the method of Benjamini and Hochberg [18]. Analysis of MDD and BPD cohorts followed the same procedure.

Raw data is stored in our database and can be made available upon request.

## Results

### Identification of Analytes Altered in Schizophrenia Patients Compared to Control Subjects

Multiplex immunoassay profiling of serum samples resulted in identification of 65 analytes which were present at significantly different levels between schizophrenia patients and controls after adjustment for cohort, age, and sex (**Table 3**). Forty-one analytes are shown with Q<0.10. The analytes showing the largest ratiometric differences included ferritin, macrophage migration inhibitory factor, carcinoembryonic antigen and haptoglobin, which were all more than 1.5-fold higher in schizophrenia patients compared to controls. Epidermal growth factor was decreased by more than 1.5 fold. Seven of these 41 analytes showed significant interactions of diagnosis with sex. These were ferritin, testosterone (total and free), alpha-1-antitrypsin, thyroxine binding globulin, interleukin (IL)-15, macrophage-derived chemokine (MDC) and angiotensin-converting enzyme.

**Table 3:** Multiplex immunoassay profiling analysis of schizophrenia and control samples across the combined cohorts.

	P	Q	R
Ferritin*	<0.001	<0.001	2.23
**Macrophage migration inhibitory factor (MIF)**	<0.001	<0.001	1.88
**Cortisol**	<0.001	<0.001	1.42
**Alpha-2-macroglobulin**	<0.001	<0.001	1.16
**Haptoglobin**	<0.001	<0.001	1.57
**Carcinoembryonic antigen**	<0.001	<0.001	1.59
Testosterone*	<0.001	<0.001	1.23
**Sortilin**	<0.001	<0.001	0.85
**Interleukin-8**	<0.001	<0.001	1.34
**Interleukin-10**	<0.001	0.001	1.11
**Epidermal growth factor**	<0.001	0.002	0.62
**Complement C3**	0.002	0.013	1.07
**Apolipoprotein C-III**	0.002	0.018	0.88
**CD40 ligand**	0.003	0.018	0.72
**Tissue inhibitor of metalloproteinases 1**	0.004	0.026	1.06
Free testosterone*	0.005	0.028	1.26
Alpha-1-antitrypsin*	0.005	0.028	1.07
**Luteinizing hormone**	0.005	0.028	1.29
**Pancreatic polypeptide**	0.006	0.028	1.42
**Apolipoprotein A-1**	0.006	0.028	0.89
**Follicle stimulating hormone**	0.007	0.035	1.27
**T cell specific protein RANTES (RANTES)**	0.008	0.036	1.17
**Prostatic acid phosphatase**	0.008	0.036	0.89
Thyroxine binding globulin*	0.010	0.040	1.08
**Progesterone**	0.010	0.041	1.18
**Resistin**	0.012	0.045	0.88
**Tumor necrosis factor receptor-like 2 (TNFR2)**	0.014	0.045	1.08
**Insulin-like growth factor-binding protein 2 (IGFBP-2)**	0.014	0.045	1.15
Interleukin-15*	0.015	0.045	1.17
Hepatocyte growth factor	0.015	0.045	1.17
**Chromogranin A**	0.015	0.045	1.34
**Serum amyloid P**	0.015	0.045	1.09
Receptor for advanced glycosylation end products (RAGE)	0.016	0.045	0.87
Macrophage-derived chemokine (MDC)*	0.016	0.045	1.09
EN-RAGE	0.017	0.048	1.26
Creatine kinase-MB	0.018	0.048	0.82
Tenascin-C	0.024	0.064	1.12
**Eotaxin**	0.025	0.065	1.15
Angiotensin converting enzyme (ACE)*	0.029	0.073	0.88
**Factor VII**	0.034	0.082	0.92
**Leptin**	0.035	0.084	0.78

P = p-value. Q = p-value adjusted for the false discovery rate. R = ratio (schizophrenia/control) using geometric means. Analytes with an asterisk showed a significant diagnosis gender interaction. Analytes in bold font have been identified in previous multiplex immunoassay profiling analyses of schizophrenia and control patients.

### Sex Specificity of Schizophrenia Biomarkers

The second part of this study was targeted at the identification of analytes with significantly different concentrations between males and females in schizophrenia (P<0.05 and Q<0.30). This resulted in identification of 16 molecules with significant diagnosis-sex interactions across the 4 cohorts. We also approximated free testosterone levels using the ratio of total testosterone to sex hormone binding globulin (SHBG) and found a significant diagnosis-sex interaction (P<0.001). We obtained qualitatively similar results using a non-parametric interaction test without covariate adjustment. For ease of interpretation, all analytes with significant interactions were categorized as “male-specific”, “female-specific” or “qualitative interaction”, depending on whether the respective abnormalities occurred only in males, only in females or in both sexes but with opposite directional changes (**Table 4**). This analysis showed that serum from male schizophrenia patients contained higher levels of ferritin, alpha-1-antitrypsin, MDC, thyroxine binding globulin, IL-15, macrophage inflammatory protein-1 alpha (MIP-1 alpha), intracellular adhesion molecule (ICAM)-1 and epithelial-derived neutrophil-activating protein (ENA)-78. Female sera contained higher levels of total and free testosterone, and lower levels of angiotensin-converting enzyme (ACE) and vascular endothelial growth factor (VEGF). We also measured the prolactin/growth hormone and insulin/growth hormone ratios as these are known to be affected in insulin resistant states, which can occur in some schizophrenia patients [19]–[21]. We found higher ratios for both of these in sera from female patients. The levels of B lymphocyte chemoattractant (BLC), prolactin, SHBG, stem cell factor (SCF) and growth hormone showed qualitative interactions with changes seen in opposite directions between male and female patients. The majority of analytes with significant overall sex-diagnosis interactions also showed similar sex-specific trends within individual cohorts (**Table S4 in the File S1**), although there was substantial variation in patterns for levels of prolactin and growth hormone.

**Table 4:** Summary of significant diagnosis-sex interactions in schizophrenia and treatment effects on the levels of analytes.

	Interaction		Males		Females		Treatment Main Effect	
	P	Q	R	P	R	P	R	P
*Male specific*								
Ferritin	0.002	0.04	2.89	<0.001	1.51	0.027	0.86	0.082
Alpha-1-antitrypsin	0.043	0.26	1.11	<0.001	1.01	0.81	0.97	0.26
MDC	0.006	0.09	1.19	<0.001	0.97	0.60		
Thyroxine binding globulin	0.039	0.25	1.13	<0.001	1.00	0.92	1.04	0.23
Interleukin-15	0.017	0.16	1.33	<0.001	0.96	0.77	0.85	0.30
MIP-1 alpha	0.016	0.16	1.13	0.003	0.99	0.54	0.97	0.28
ICAM-1	0.019	0.16	1.11	0.008	0.97	0.45	**1.14**	**0.006**
ENA-78	0.048	0.27	1.18	0.027	0.93	0.45	1.05	0.16
*Female specific*								
Testosterone	<0.001	0.03	1.10	0.062	1.51	<0.001		
Free testosterone	<0.001	0.002	0.94	0.42	2.01	<0.001		
ACE	0.015	0.16	0.99	0.87	0.74	0.002	**1.21**	**<0.001**
VEGF	0.034	0.25	1.03	0.65	0.86	0.011	1.00	0.93
Prolactin/growth hormone	0.007	0.10	0.66	0.17	2.36	0.008	**3.94**	**0.015**
Insulin/growth hormone	0.038	0.25	0.72	0.42	2.54	0.015	1.44	0.47
*Qualitative interaction*								
BLC	<0.001	0.03	1.73	0.009	0.59	0.035	1.45	0.17
Prolactin	0.038	0.25	0.90	0.32	1.33	0.062	**2.54**	**<0.001**
SHBG	0.001	0.04	1.15	0.030	0.74	0.023	**0.81**	**0.008**
Stem cell factor	0.012	0.16	1.06	0.17	0.88	0.035	1.06	0.10
Growth hormone	0.040	0.25	1.41	0.21	0.58	0.079	0.64	0.35

Analytes were grouped into male or female specific sets accordingly. P = p-value. Q = p-value adjusted for the false discovery rate. R = ratio (schizophrenia/control; follow-up/baseline) using geometric means. Significant treatment effects are shown in bold. MDC = macrophage-derived chemokine. MIP-1 alpha = macrophage inflammatory protein-1 alpha. ICAM-1 = intracellular adhesion molecule-1. ENA-78 = epithelial-derived neutrophil-activating protein-78. ACE = angiotensin-converting enzyme. VEGF = vascular endothelial growth factor. BLC = B lymphocyte chemoattractant. SHBG = sex hormone binding globulin.

### Sex Specificity of Major Depressive Disorder and Bipolar Disorder Markers

In order to examine molecular sex differences in other psychiatric diseases, we calculated sex-diagnosis interactions for the same set of analytes in groups of treated MDD and BPD patients and matched controls. We found 3 molecules with significant sex-diagnosis interactions in MDD that included higher levels of ferritin and lower levels of immunoglobulin A (IgA) in male MDD patients, along with lower growth hormone concentrations in females (**Table S5 in the File S1)**. In BPD, 7 molecules were found with sex-specific signatures, with lower male analyte levels of insulin-like growth factor binding protein 2 (IGFBP2) and myoglobin, and higher granulocyte-colony stimulating factor (GCSF) and haptoglobin levels (**Table S6 in the File S1)**. As in MDD, female BPD patients had lower serum levels of growth hormone. Chromogranin A and AXL receptor tyrosine kinase concentrations showed qualitative interactions in BPD.

### Sex Specific Associations between Analytes and PANSS Ratings

In the next part of the study, we investigated the relationship of positive, negative, and total PANSS ratings in schizophrenia patients using the panel of sex-specific biomarkers. We found that 5 molecules showed sex differences in their associations with symptom severity (**Table 5**). In female patients, the inflammation-related analytes alpha-1-antitrypsin, BLC and IL-15 showed negative associations with positive and total PANSS, positive PANSS, and negative and total PANSS, respectively. In male patients, the hormones prolactin and testosterone were negatively associated with positive PANSS ratings.

**Table 5:** Summary of sex-specific associations with positive, negative, and total PANSS score ratings.

	A1AT	IL-15	BLC	PRL	TT
**Interaction Positive PANSS**	0.012	0.039	0.032	0.037	0.018
**Male**				−0.39 (0.017)	−0.37 (0.010)
**Female**	−0.35 (0.028)	−0.47 (0.010)	−0.23 (0.128)		
**Interaction Negative PANSS**			0.004		
**Male**					
**Female**			−0.47 (0.001)		
**Interaction Total PANSS**	0.018		0.003		
**Male**					
**Female**	−0.28 (0.047)		−0.32 (0.004)		

Only molecules with significant diagnosis-sex interactions were investigated. A1AT = alpha-1-antitrypsin. BLC = B lymphocyte chemoattractant. PRL = prolactin. TT = total testosterone. The tabulated values are the average correlation coefficient from Fisher’s z transformation from linear model and (P-values).

### Sex Specific Effects on Analytes Following Schizophrenia Treatment

To test for sex specific effects of antipsychotic treatment, serum was analyzed from 33 antipsychotic naive schizophrenia patients before and after 6 weeks of inpatient treatment (**Table 2**). Of the analytes with significant diagnosis-sex interactions described above, antipsychotic treatment induced significant changes in the levels of 4 molecules (ICAM-1, ACE, prolactin and SHBG) and sex-specific changes were found for two analytes (testosterone and MDC). The largest change was observed for prolactin, which was increased 2.54-fold following treatment.

We tested the same serum samples for effects of treatment on all 95 analytes measured using the multiplex immunoassay platform. This resulted in identification of 5 analytes [testosterone, serum glutamic oxaloacetic transaminase (SGOT), follicle stimulating hormone (FSH), IL-13, and MDC] which showed differential changes, dependent on sex (**Table 6**). Levels of SGOT were increased and IL-13 was decreased specifically in females while free testosterone was increased in males. MDC levels were significantly elevated in both sexes, but greater changes were seen in females.

**Table 6:** Summary of analytes with sex-specific treatment effects.

	Interaction	Males		Female	
	P	R	P	R	P
Testosterone	0.003	1.12	0.11	0.84	0.36
Serum glutamic oxaloacetictransaminase	0.006	0.93	0.27	1.34	0.032
Free testosterone	0.009	1.37	0.004	1.07	0.80
Follicle stimulating hormone	0.021	1.01	0.84	0.62	0.10
Interleukin-13	0.026	1.01	0.79	0.87	0.012
Macrophage-derived chemokine	0.039	1.14	0.003	1.27	<0.001

P = p-value. R = ratio (follow-up/baseline) using geometric means.

## Discussion

This is the first multiplexed immunoassay study to address the question of whether sex-specific molecular differences can be identified in the circulation of schizophrenia patients. The first phase of the study was aimed at detection of serum analytes which were present at significantly different levels in schizophrenia patients compared to controls. This resulted in identification of 41 analytes, 33 of which have identified in previous multiplex immunoassay profiling studies of schizophrenia and control subjects [12], [13], [22], [23]. As described previously, these analytes represented hormonal (cortisol, luteinizing hormone, follicle-stimulating hormone, pancreatic polypeptide, progesterone, resistin, chromogranin A and leptin); growth factor (sortilin, epidermal growth factor, insulin-like growth factor binding protein 2, hepatocyte growth factor); inflammation (macrophage migration inhibitory factor, alpha-2-macroglobulin, haptoglobin, carcinoembryonic antigen, interleukin-8, interleukin-10, complement C3, CD40 ligand, tissue inhibitor of metalloproteinases 1, alpha-1-antitrypsin, RANTES, tumour necrosis factor receptor 2, serum amyloid P and eotaxin); and other (apolipoprotein CIII, apolipoprotein A1, prostatic acid phophatase) pathways. Combining multiple cohorts, increasing power and reliability of findings, resulted in the identification of 7 novel molecules associated with schizophrenia in this study. Three of these molecules showed sex-specific changes in schizophrenia.

The most novel outcome of the current study was the finding that 16 molecules showed significant sex-diagnosis interactions. Most of these molecules have been associated previously with schizophrenia, albeit not in a manner stratified by sex. In a previous study, we identified reproducible changes in alpha-1-antitrypsin, ferritin, intracellular adhesion molecule (ICAM)-1, MDC, prolactin, testosterone and VEGF [12]. The current results suggest that at least some of these differences could have been driven by specific molecular abnormalities in either male or female schizophrenia patients. In line with the numerous sex differences reported in schizophrenia, our findings included alterations in the levels of several hormones. Higher levels of free and total testosterone in female patients and concomitant sex differences in SHBG concentrations were among the most statistically significant findings. Disparity in the levels of testosterone have been associated with normal sex differences of cerebral activation during mental rotation and these patterns have been shown to be reversed in schizophrenia [24]. Mendrek *et al.* have hypothesized that this reversal of sex dimorphism is due to alterations in sex hormone levels in both males and females during neurodevelopment at the organization and activation stages, consistent with the timing of onset in schizophrenia in adolescence or early adulthood [25]. The current study puts this finding in the context of co-occurring sexually dimorphic molecular changes. SHBG binds to sex hormones like testosterone, affecting their bioavailability to target cells. This protein was decreased in female schizophrenia patients in parallel with increased free and total testosterone and differences in prolactin and growth hormone levels. Decreased SHBG has been reported in female schizophrenia patients after treatment with atypical antipsychotics, in association with increased prolactin levels [26], [27].

Though insulin resistance was not directly measured in this study, we determined the balance between insulin, growth hormone, and prolactin as an indirect indication of this phenomenon. Previous studies have suggested that 30–50% of first onset schizophrenia patients show signs of impaired insulin signalling [28]. Our finding of higher prolactin/growth hormone and insulin/growth hormone ratios in female first onset schizophrenia patients suggests that this effect could also be sex-specific. Circulating prolactin improves glucose homeostasis by increasing insulin action and secretion [19]. Conversely, high levels of growth hormone can result in insulin antagonistic effects including insulin resistance, stimulation of lipolysis, and inhibition of glucose transport [20]. Studies using growth hormone and growth hormone receptor knockout mice found increased insulin sensitivity and higher prolactin levels, as well as increased life expectancy compared to wild type mice [21]. Taken together, these findings suggest that female schizophrenia patients may show signs of better insulin signalling, which may afford some protection against development of more severe psychiatric symptoms and disease associated deficits.

Finally, molecular differences were also found specifically in male patients and these were mainly related to pathways involved in the inflammatory response. We found higher levels of ferritin in males with schizophrenia, an acute phase reactant protein elevated in the course of many diseases. Higher levels of this transport protein have also been associated with a younger age of onset in Alzheimer’s and Parkinson’s disease [29]. We found several additional analytes related to acute phase and the inflammatory response present at higher levels only in male patients. This raises the possibility that some aspects of the widely-reported immunological abnormalities in schizophrenia may be specific for males. However, other inflammation-related molecules including alpha-1-antitrypsin, IL-15 and BLC were related to positive, negative, and total symptom severity in females. Taken together, these findings suggest that different components of the inflammatory and immune responses may behave differently in male and female patients with schizophrenia.

We were unable to evaluate precisely the specificity of these results to schizophrenia by comparison with other psychiatric illnesses due to the rarity of first onset, drug naive patients. However, we examined sex-diagnosis interactions for the same analytes in a combination of treated, drug free, and drug naive MDD and treated BPD patients. We found an overlap in the sex-specific signature for growth hormone in both illnesses and also for ferritin in MDD. A study of sex-specific serum biomarkers using the same analytes in adults with Asperger syndrome (AS; AS = 45, control = 50) revealed elevated levels of free testosterone specifically in females and similar patterns of sex differences in growth hormone and SHBG concentrations compared to the current study of schizophrenia patients and controls [30]. These findings reveal similar and distinct sex-specific molecular patterns in psychiatric disease. Larger and better characterized cohorts of first onset, drug naive major depression, bipolar disorder, and other psychiatric disorder patients would be required to adequately evaluate the specificity of the sex-specific molecular phenotype of schizophrenia.

The present findings suggest that sex-specific differences occur in the response and role of hormones, growth factors, cardiovascular and inflammation pathways in schizophrenia. However, there are several limitations which will require further work. For example, larger cohorts for replication and better characterized samples will be needed. Fewer female patient samples were available, reducing the power to detect molecular differences in this group. Larger cohort sizes and more female patient samples would facilitate studies of the effects of age on sex-specific molecular signatures of schizophrenia as levels of estrogen in females decline. In addition, we cannot account for unrecorded potential confounding factors such as BMI, tobacco, and cannabis consumption in some cohorts. Information on other prescription drug use including contraceptive medication and other variables like socioeconomic status, fasting status, blood pressure, menopausal status, and phase of menstrual cycle were also unavailable for all cohorts. However, the size and number of cohorts in this study are likely to compensate for potential confounders and provides a naturalistic clinical scenario. Finally, stricter significance cut-offs using adjusted p-values would have lead to different results and interpretation of this study, though internal replication between cohorts showed consistent patterns for most analytes.

A small scale longitudinal study showed that antipsychotic treatment led to significant sex-specific changes in testosterone and MDC, providing further evidence of the potential effect of sex on the role of these molecules in schizophrenia. We also found SGOT increased specifically in females using antipsychotics, which may be related to weight gain [31]. In addition to the metabolic side effects of antipsychotics, cardiovascular mortality and hypertension are more prevalent in patients with psychotic illness [32]. These are potentially associated with the elevation in concentrations of ACE and ICAM-1 we found in patients over the course of antipsychotic treatment. Previous investigations have also reported increased prolactin and decreased SHBG levels following atypical antipsychotic administration and these findings are replicated here [26], [27], [33], [34]. Larger follow-up studies will be necessary to investigate the sex-specific modulation of schizophrenia biomarker candidates. Further exploration is also warranted into the mechanisms by which these sexually distinct molecular phenotypes in schizophrenia arise. This may lead to deeper insights into the well established sex differences in the clinical manifestation and course of schizophrenia which, in turn, could lead to the development of novel treatment approaches for improved patient outcomes.

## Supporting Information

File S1 **Table S1-Table S6.** Table S1. Major depressive disorder (MDD) patient demographics. Table S2. Bipolar disorder (BPD) patient demographics. Table S3. List of 95 analytes measured using the HumanMAP® multiplex immunoassay platform and included in the comparison across all cohorts. Table S4. Summary of sex-specific changes in analyte levels within cohorts. Table S5. Summary of analyte levels with significant diagnosis-sex interactions in major depressive disorder (MDD). Table S6. Summary of analyte levels with significant diagnosis-sex interactions in bipolar disorder (BPD).
(DOC)
