# Supplementary material for: Distinct Molecular Phenotypes in Male and Female Schizophrenia Patients
Source: PLoS One. 2013 Nov 11;8(11):e78729. doi: 10.1371/journal.pone.0078729 (PMC3823995; doi:10.1371/journal.pone.0078729)
Supplement: File S1 — Table S1-Table S6. Table S1. Major depressive disorder (MDD) patient demographics. Table S2. Bipolar disorder (BPD) patient demographics. Table S3. List of 95 analytes measured using the HumanMAP® multiplex immunoassay platform and included in the comparison across all cohorts. Table S4. Summary of sex-specific changes in analyte levels within cohorts. Table S5. Summary of analyte levels with significant diagnosis-sex interactions in major depressive disorder (MDD). Table S6. Summary of analyte levels with significant diagnosis-sex interactions in bipolar disorder (BPD). (DOC) [file pone.0078729.s001.doc]

**Supplementary Table 1.** Major depressive disorder (MDD) patient demographics. Values are shown as mean ± sd. Tobacco and cannabis use is displayed as (yes/no/NA) and age is in years. M = male. F = female. N = number. BMI = body mass index (kg/m^2^). ECT = electroconvulsive therapy. Cohorts MDD1 and 2 were from University of Cologne Department of Psychiatry, Germany; Cohorts MDD3 and 4 were from University of Magdeburg, Germany; and Cohort MDD5 was from University of Würzburg, Germany. Patients from University of Würzburg with medical conditions such as type 2 diabetes mellitus, hypertension, endocrine abnormalities, cardiovascular or autoimmune diseases were not excluded from this study. Most patients were not experiencing the first episode of illness and were using psychiatric medication.

|  | Cohort MDD1 | | Cohort MDD2 | | Cohort MDD3 | | Cohort MDD4 | | Cohort MDD5 | |
| --- | --- | --- | --- | --- | --- | --- | --- | --- | --- | --- |
| Sex | M | F | M | F | M | F | M | F | M | F |
| Patients N | 32 | 44 | 11 | 22 | 18 | 29 | 4 | 18 | 7 | 11 |
| Controls N | 32 | 44 | 11 | 22 | 18 | 29 | 4 | 18 | 7 | 11 |
| Patients age | 42.9±12 | 40.5±11 | 38.3±11 | 40.7±13 | 44.2±14 | 39.2±14 | 42.8±8 | 35.8±11 | 50.9±4 | 48.2±12 |
| Controls age | 37.5±16 | 39.7±12 | 38.5±11 | 40.2±14 | 43.3±13 | 38.5±13 | 42.5±8 | 34.1±10 | 47.7±7 | 44.4±12 |
| Patients BMI |  |  | 28.9±4 | 27.1±5 | 25.2±3 | 23.4±4 | 24.5±6 | 24.3±7 |  |  |
| Controls BMI |  |  |  |  | 25.4±3 | 23.1±3 | 24.7±3 | 22.1±3 |  |  |
| Patients tobacco |  |  | 4/4/3 | 6/14/2 | 7/11/0 | 8/21/0 | 4/0/0 | 8/10/0 | 4/3/0 | 3/8/0 |
| Controls tobacco |  |  |  |  | 3/15/0 | 3/26/0 | 2/2/0 | 3/15/0 | 0/7/0 | 2/8/1 |
| Patients cannabis |  |  | 4/6/1 | 6/15/1 | 0/15/3 | 0/28/1 | 1/3/0 | 1/17/0 |  |  |
| Controls cannabis |  |  |  |  | 0/17/1 | 0/25/4 | 0/4/0 | 0/18/0 |  |  |
| Psychiatric medication | Mixture ECT/ antidepressant use/ drug free | | Antidepressant and antipsychotic medication use | | Antidepressant free/naive; first episode | | Mixture antidepressant use/drug naive | | Mixture antidepressant/ antipsychotic use | |

**Supplementary Table 2.** Bipolar disorder (BPD) patient demographics. Values are shown as mean ± sd. Tobacco and cannabis use is displayed as (yes/no/NA) and age is in years. M = male. F = female. N = number. BMI = body mass index (kg/m^2^). Cohorts BPD1 and 2 were from University of Cologne Department of Psychiatry, Germany; Cohort BPD3 was from the University of Muenster, Germany; Cohort BPD4 was from the Erasmus Medical Centre, the Netherlands; and Cohorts BPD5-7 were from of Würzburg, Germany. Patients from University of Würzburg with medical conditions such as type 2 diabetes mellitus, hypertension, endocrine abnormalities, cardiovascular or autoimmune diseases were not excluded from this study. Patients across cohorts were diagnosed with a mixture of BPD types and were experiencing a variety of mood states. BPD patients in all cohorts were using psychiatric medication.

|  | Cohort BPD1 | | Cohort BPD2 | | Cohort BPD3 | | Cohort BPD4 | | Cohort BPD5 | | Cohort BPD6 | | Cohort BPD7 | |
| --- | --- | --- | --- | --- | --- | --- | --- | --- | --- | --- | --- | --- | --- | --- |
| Sex | M | F | M | F | M | F | M | F | M | F | M | F | M | F |
| Patients N | 4 | 3 | 5 | 8 | 11 | 10 | 7 | 3 | 9 | 12 | 3 | 3 | 5 | 9 |
| Controls N | 4 | 3 | 5 | 8 | 11 | 10 | 7 | 3 | 9 | 12 | 3 | 3 | 5 | 9 |
| Patients age | 23.8±3 | 29.3±5 | 45.4±13 | 40.9±9 | 45.5±11 | 43.3±14 | 26.1±10 | 33.3±15 | 44.1±11 | 44.1±13 | 42±15 | 51±22 | 47.6±9 | 45.6±13 |
| Controls age | 25.5±2 | 29±7 | 39.6±10 | 39.9±9 | 38.5±11 | 43.5±14 | 24.4±6 | 28.3±3 | 35.6±11 | 34.4±12 | 39.7±13 | 49.3±20 | 48.4±8 | 45.7±13 |
| Patients BMI | 22.6±1 | 22.4±2 |  |  | 34±17 | 27.4±6 |  |  | 28.8±5 | 24.7±7 |  |  |  |  |
| Controls BMI | 22.6±1 | 20.9±2 |  |  |  |  |  |  | 25.1±4 | 24.4±4 |  |  |  |  |
| Patients tobacco | 1/0/3 | 2/1/0 | 3/2/0 | 6/2/0 | 2/9/0 | 4/6/0 | 7/0/0 | 3/0/0 | 5/4/0 | 5/7/0 | 0/3/0 | 2/1/0 | 0/5/0 | 0/9/0 |
| Controls tobacco | 3/1/0 | 2/1/0 | 1/4/0 | 1/7/0 |  |  |  |  | 2/7/0 | 5/7/0 | 0/3/0 | 1/2/0 | 0/5/0 | 1/7/1 |
| Patients cannabis | 1/0/3 | 3/0/0 | 0/5/0 | 0/8/0 | 6/5/0 | 2/8/0 | 5/2/0 | 1/2/0 |  |  |  |  |  |  |
| Controls cannabis | 0/3/1 | 1/1/1 | 0/5/0 | 1/6/1 |  |  |  |  |  |  |  |  |  |  |

**Supplementary Table 3.** List of 95 analytes measured using the HumanMAP® multiplex immunoassay platform and included in the comparison across all cohorts.

Adiponectin

Alpha-1-Antitrypsin (A1AT)

Alpha-2-Macroglobulin (A2Macro)

Alpha-Fetoprotein (AFP)

Angiopoietin-2 (ANG-2)

Angiotensin-Converting Enzyme (ACE)

Angiotensinogen

Apolipoprotein A-I (Apo A-I)

Apolipoprotein C-III (Apo C-III)

Apolipoprotein H (Apo H)

Apolipoprotein(a) (Lp(a))

AXL Receptor Tyrosine Kinase (AXL)

B Lymphocyte Chemoattractant (BLC)

Beta-2-Microglobulin (B2M)

Brain-Derived Neurotrophic Factor (BDNF)

Cancer Antigen 19-9 (CA 19-9)

Carcinoembryonic Antigen (CEA)

CD 40 Antigen (CD40)

CD40 Ligand (CD40-L)

Chemokine CC-4 (HCC-4)

Chromogranin A (CgA)

Complement C3 (C3)

Cortisol

C-Reactive Protein (CRP)

Creatine Kinase-MB (CK-MB)

EN-RAGE

Eotaxin-1

Epidermal Growth Factor (EGF)

Epidermal Growth Factor Receptor (EGFR)

Epithelial-Derived Neutrophil Activating Protein 78 (ENA-78)

Factor VII

FASLG Receptor (FAS)

Ferritin (FRTN)

Follicle-Stimulating Hormone (FSH)

Glutathione S-Transferase alpha (GST-alpha)

Granulocyte Colony-Stimulating Factor (G-CSF)

Growth Hormone (GH)

Growth-Regulated alpha protein (GRO-alpha)

Haptoglobin

Heparin-Binding EGF-Like Growth Factor (HB-EGF)

Hepatocyte Growth Factor (HGF)

Immunoglobulin A (IgA)

Immunoglobulin E (IgE)

Immunoglobulin M (IgM)

Insulin

Insulin-like Growth Factor-Binding

Protein 2 (IGFBP-2)

Intercellular Adhesion Molecule 1 (ICAM-1)

Interleukin-1 receptor antagonist (IL-1ra)

Interleukin-10 (IL-10)

Interleukin-13 (IL-13)

Interleukin-15 (IL-15)

Interleukin-16 (IL-16)

Interleukin-18 (IL-18)

Interleukin-3 (IL-3)

Interleukin-7 (IL-7)

Interleukin-8 (IL-8)

Leptin

Luteinizing Hormone (LH)

Macrophage Inflammatory

Protein-1 alpha (MIP-1 alpha)

Macrophage Inflammatory

Protein-1 beta (MIP-1 beta)

Macrophage Migration

Inhibitory Factor (MIF)

Macrophage-Derived Chemokine (MDC)

Matrix Metalloproteinase-3 (MMP-3)

Monocyte Chemotactic Protein 1 (MCP-1)

Myeloperoxidase (MPO)

Myoglobin

Pancreatic Polypeptide (PPP)

Plasminogen Activator Inhibitor 1 (PAI-1)

Platelet-Derived Growth Factor BB (PDGF-BB)

Progesterone

Prolactin (PRL)

Prostatic Acid Phosphatase (PAP)

Pulmonary and Activation-

Regulated Chemokine (PARC)

Receptor for Advanced Glycosylation End Products (RAGE)

Resistin

Serum Amyloid P-Component (SAP)

Serum Glutamic Oxaloacetic Transaminase (SGOT)

Sex Hormone-Binding Globulin (SHBG)

Sortilin

Stem Cell Factor (SCF)

Superoxide Dismutase 1, Soluble (SOD-1)

T-Cell-Specific Protein RANTES (RANTES)

Tenascin-C (TN-C)

Testosterone, Total (TT)

Thrombopoietin

Thrombospondin-1 (TSP-1)

Thyroid-Stimulating Hormone (TSH)

Thyroxine-Binding Globulin (TBG)

Tissue Inhibitor of Metalloproteinases 1 (TIMP-1)

TNF-Related Apoptosis-Inducing Ligand Receptor 3 (TRAIL-R3)

Tumor Necrosis Factor alpha (TNF-alpha)

Tumor Necrosis Factor Receptor-Like 2 (TNFR2)

Vascular Cell Adhesion Molecule-1 (VCAM-1)

Vascular Endothelial Growth Factor (VEGF)

von Willebrand Factor (vWF)

**Supplementary Table 4.** Summary of sex-specific changes in analyte levels within cohorts. Analytes with significant overall sex-diagnosis interactions are shown (from Table 3). Significant differences between schizophrenia patients and controls are indicated in bold. R/R1-4 = overall/Cohort 1-4 ratio (schizophrenia/control) using geometric means. MDC = macrophage-derived chemokine. MIP-1 alpha = macrophage inflammatory protein-1 alpha. ICAM-1 = intracellular adhesion molecule-1. ENA-78 = epithelial-derived neutrophil-activating protein-78. ACE = angiotensin-converting enzyme. VEGF = vascular endothelial growth factor. BLC = B lymphocyte chemoattractant. SHBG = sex hormone binding globulin.

|  | **Males** | | | | | **Females** | | | | |
| --- | --- | --- | --- | --- | --- | --- | --- | --- | --- | --- |
|  | **R** | **R1** | **R2** | **R3** | **R4** | **R** | **R1** | **R2** | **R3** | **R4** |
| *Male specific* |  |  |  |  |  |  |  |  |  |  |
| Ferritin | **2.89** | **1.64** | **4.79** | 1.37 | **6.00** | **1.51** | 1.06 | 1.78 | 1.61 | **3.35** |
| Alpha-1-antitrypsin | **1.12** | **1.24** | 1.04 | 1.08 | 1.18 | 1.01 | 0.99 | 0.97 | **1.27** | 0.86 |
| MDC | **1.19** | 1.04 | **1.31** | 1.06 | 1.37 | 0.97 | 1.01 | 0.93 | 1.11 | 0.76 |
| Thyroxine binding globulin | **1.13** | **1.12** | **1.15** | 1.05 | 1.22 | 1.00 | 0.99 | 0.95 | 1.14 | 0.94 |
| Interleukin-15 | **1.33** | 1.10 | **1.35** | 1.31 | **2.23** | 0.96 | 0.97 | 1.08 | 0.92 | 0.87 |
| MIP-1 alpha | **1.13** | 0.91 | **1.32** | 0.99 | 1.40 | 0.99 | **0.85** | 1.15 | 1.00 | 1.15 |
| ICAM-1 | **1.11** | 0.93 | **1.17** | 1.23 | 1.24 | 0.97 | 0.90 | 0.98 | 1.08 | 1.11 |
| ENA-78 | **1.18** | 1.14 | **1.25** | 1.05 | 1.27 | 0.93 | 0.94 | 1.22 | 0.65 | 0.97 |
| *Female specific* |  |  |  |  |  |  |  |  |  |  |
| Testosterone | 1.10 | 1.09 | **1.20** | 0.95 | 1.01 | **1.51** | **1.44** | **1.47** | **2.04** | **1.45** |
| Free testosterone | 0.94 | 1.15 | 0.90 | 0.86 | 0.76 | **2.01** | **2.24** | 1.40 | **2.16** | **2.56** |
| ACE | 0.99 | 0.98 | 0.93 | 1.18 | 0.97 | **0.74** | 0.80 | **0.51** | 0.87 | 0.83 |
| VEGF | 1.03 | 0.87 | 1.13 | 0.88 | **1.43** | **0.86** | **0.85** | 0.85 | 1.15 | **0.65** |
| Prolactin/growth hormone | 0.66 | 0.59 | 0.57 | 0.38 | 4.06 | **2.36** | 1.60 | **8.12** | 1.22 | 3.35 |
| Insulin/growth hormone | 0.72 | 0.77 | 0.50 | 0.55 | 4.79 | **2.55** | 0.80 | **7.92** | 3.84 | **10.13** |
| *Qualitative interaction* |  |  |  |  |  |  |  |  |  |  |
| BLC | **1.73** | 0.76 | **3.14** | 1.36 | 2.21 | **0.59** | **0.35** | 0.94 | 0.88 | 1.04 |
| Prolactin | 0.90 | 1.06 | 0.81 | 0.76 | 1.17 | 1.33 | **2.16** | 1.26 | 0.74 | 0.63 |
| SHBG | **1.15** | 0.92 | **1.33** | 1.09 | 1.33 | **0.74** | **0.65** | 0.93 | 0.99 | 0.55 |
| Stem cell factor | 1.06 | 1.00 | 1.12 | 0.98 | 1.18 | **0.88** | 0.87 | 0.96 | 0.76 | 0.98 |
| Growth hormone | 1.41 | 1.98 | 1.41 | 2.01 | 0.29 | 0.58 | **1.49** | **0.15** | 0.61 | 0.19 |

**Supplementary Table 5.** Summary of analyte levels with significant diagnosis-sex interactions in major depressive disorder (MDD). Analytes were grouped into male or female specific sets accordingly. P = p-value. Q = p-value adjusted for the false discovery rate. R = ratio (schizophrenia/control) using geometric means.

|  | **Interaction** | | **Males** | | **Females** | |
| --- | --- | --- | --- | --- | --- | --- |
|  | **P** | **Q** | **R** | **P** | **R** | **P** |
| *Male specific* | | | | | | |
| Ferritin | 0.003 | 0.154 | 3.23 | <0.001 | 1.78 | <0.001 |
| Immunoglobulin A | 0.006 | 0.181 | 0.73 | <0.001 | 0.95 | 0.360 |
| *Female specific* | | | | | | |
| Growth hormone | <0.001 | 0.007 | 1.10 | 0.873 | 0.25 | <0.001 |

**Supplementary Table 6.** Summary of analyte levels with significant diagnosis-sex interactions in bipolar disorder (BPD). Analytes were grouped into male or female specific sets accordingly. P = p-value. Q = p-value adjusted for the false discovery rate. R = ratio (schizophrenia/control) using geometric means.

|  | **Interaction** | | **Males** | | **Females** | |
| --- | --- | --- | --- | --- | --- | --- |
|  | **P** | **Q** | **P** | **FC** | **P** | **FC** |
| *Male specific* | | | | | | |
| Insulin-like growth factor-binding protein 2 | 0.004 | 0.125 | 0.003 | 0.75 | 0.412 | 1.12 |
| Granulocyte-colony stimulating factor | 0.005 | 0.125 | 0.025 | 1.43 | 0.167 | 0.87 |
| Haptoglobin | 0.015 | 0.231 | 0.004 | 1.75 | 0.657 | 0.94 |
| Myoglobin | 0.005 | 0.125 | 0.010 | 0.80 | 0.292 | 1.12 |
| *Female specific* | | | | | | |
| Growth hormone | 0.005 | 0.125 | 0.471 | 0.76 | <0.001 | 0.17 |
| *Qualitative interaction* | | | | | | |
| Chromogranin A | 0.011 | 0.204 | 0.134 | 0.73 | 0.085 | 1.78 |
| AXL receptor tyrosine kinase | 0.020 | 0.272 | 0.085 | 0.86 | 0.261 | 1.08 |
